# Remnant Cholesterol Inflammatory Index and New-Onset Diabetes in Middle-Aged and Older Adults: Evidence From Prospective Surveys of Chinese and UK Populations

**DOI:** 10.1155/jdr/1579313

**Published:** 2025-11-28

**Authors:** Nan Chen, Ajuan Gong, Xiaomin Huang, Nuojin Wang, Tianrong Pan, Xiaoyu Pan

**Affiliations:** Department of Endocrinology, The Second Affiliated Hospital of Anhui Medical University, Hefei, Anhui, China

**Keywords:** Chinese Health and Retirement Longitudinal Study, diabetes, English Longitudinal Study of Ageing, high sensitivity C-reactive protein, remnant cholesterol inflammatory index

## Abstract

**Aim:**

The aim of this study was to investigate the relationship between remnant cholesterol inflammatory index (RCII) and new-onset diabetes mellitus in middle-aged and elderly populations in China and United Kingdom.

**Methods:**

The total number of participants included in this study was 9946, comprising members of the Chinese Health and Retirement Longitudinal Study (CHARLS) cohort and the English Longitudinal Study of Ageing (ELSA) cohort. A comparison was made of the baseline characteristics of the two cohorts. Subsequently, a cox regression analysis was performed on the risk factors for diabetes. Subgroup analyses were conducted to explore the potential for effect modification across diverse subgroups.

**Results:**

The application of RCII quartile analyses revealed that the risk observed in the highest quartile (Q4) within the ELSA cohort was 8.49 times higher than that recorded in the lowest quartile (Q1). This finding was particularly significant for males. Similarly, the risk in the CHARLS cohort was 3.1 times higher than that in Q1. Following multi-model adjustment, the risk of diabetes exhibited a progressive increase from the second to the fourth quartile of RCII levels in both cohorts, with all associations demonstrating statistical significance. For each 1 kg/m^2^ increase in body mass index (BMI), the risk of diabetes increased by 13% in the ELSA cohort and by 15% in the CHARLS cohort. Subgroup analyses revealed that within the CHARLS cohort, the association between RCII and diabetes was more pronounced among non-obese individuals, whereas in the ELSA cohort, this link was more evident among middle-aged adults.

**Conclusions:**

Elevated RCII levels have been demonstrated to be significantly associated with an increased risk of future diabetes in middle-aged and older adults.

## 1. Introduction

The global prevalence of diabetes mellitus is a major public health concern, which is further exacerbated by population ageing and changes in lifestyle. The demographic group comprising middle-aged and elderly individuals constitutes a high prevalence group for diabetes mellitus. According to the statistics of the International Diabetes Federation (IDF), the prevalence of diabetes mellitus among middle-aged and elderly people has been increasing on an annual basis on a global scale. This has resulted in a significant burden being placed on personal health, family economy and social healthcare resources [1]. In China, the issue of diabetes mellitus is becoming increasingly salient, particularly in relation to the country's rapidly ageing population. Diabetes has been shown to trigger a series of symptoms related to hyperglycaemia, as well as leading to a range of serious chronic complications. These include cardiovascular disease, nephropathy, neuropathy and retinopathy, among others. These complications have been demonstrated to have a significant impact on the quality of life of patients, and can even pose a threat to their lives [2–4]. Consequently, conducting a thorough investigation into the pathogenesis of diabetes, in addition to the identification of effective early prediction indicators and intervention targets, is of paramount importance in the prevention and management of diabetes in middle-aged and elderly individual.

The remnant cholesterol inflammatory index (RCII) represents an emerging index of lipid-related inflammation, which has attracted increasing attention from researchers in recent years. It is hypothesised that the RCII may be capable of reflecting lipid metabolism disorders and inflammation more comprehensively through its consideration of remnant cholesterol and inflammatory factors. Residual cholesterol is defined as an intermediate product of the metabolism of lipoproteins rich in triglycerides (TRLs). These include very low-density lipoprotein (VLDL) remnants, intermediate-density lipoprotein (IDL) and so on, and elevated levels of this product are closely associated with the risk of atherosclerotic cardiovascular disease [5–7]. Furthermore, inflammation has been demonstrated to play a pivotal role in the pathogenesis of this condition. It has been observed that a chronic low-grade inflammatory state is capable of inducing insulin resistance and impaired pancreatic *β*-cell function, which, in turn, promotes the development of diabetes mellitus. High-sensitivity C-reactive protein (hsCRP) has been identified as a sensitive marker reflecting chronic low-grade inflammation. Elevated levels of this protein have been observed in response to an inflammatory stimulus. A mounting body of research has indicated a strong correlation between chronic inflammation and insulin resistance, as well as glucose metabolism disorders [8–10]. However, the specific mechanism by which hsCRP contributes to the development of diabetes in middle-aged and elderly individuals remains to be fully elucidated. The combination of residual cholesterol and hsCRP in RCII offers a novel approach for evaluating the risk of diabetes development. This combination has the potential to serve as a biomarker for predicting the onset of diabetes in middle-aged and elderly individuals.

As demonstrated in previous studies, elevated RCII levels have been found to be associated with all-cause mortality and the occurrence of stroke [11, 12]. However, studies addressing the relationship between RCII and new-onset diabetes in middle-aged and older adults are currently unavailable. It is evident that exhaustive analyses employing substantial, prospective and representative databases can yield more precise insights regarding the underlying relationship between the two. These analyses provide a scientific foundation for the early prevention and intervention measures that are imperative in the management of diabetes mellitus. The China Health and Retirement Longitudinal Study (CHARLS) and the English Longitudinal Study of Ageing (ELSA) represent two extensively representative, large-scale, multidimensional prospective cohort studies, which have collected a substantial amount of health-related information, lifestyle data and biological samples from middle-aged and elderly individuals in China and United Kingdom, respectively, thus creating distinctive conditions for conducting such studies. It is hypothesised that a comprehensive exploration of the relationship between RCII and new-onset diabetes mellitus in middle-aged and elderly people can be achieved by analysing these two databases in the context of different populations. This exploration has the potential to enrich the theoretical research on the pathogenesis of diabetes mellitus and provide strong evidence to support the development of diabetes mellitus prevention strategies that are suitable for middle-aged and elderly people in different regions. The results of this research could be of great value for application in public health and clinical practice.

## 2. Method

### 2.1. Study Population

**Figure 1:**
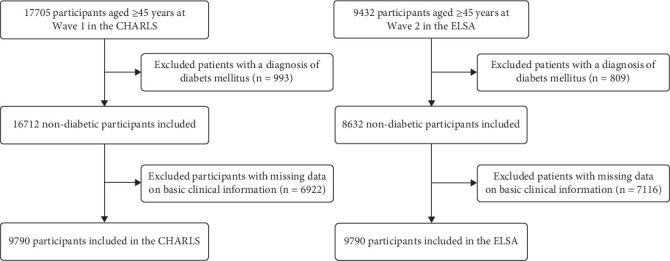
Flowchart of the population included in our study.

In this study, the data of middle-aged and older adults from the CHARLS and ELSA databases was screened, with the objective of obtaining a sample that is representative of the target population and that meets the study's requirements. This approach was taken to ensure the accuracy and reliability of the results. CHARLS is a long-term follow-up survey in China to study the health, retirement and economic status of elderly Chinese [13]. In the CHARLS database, the following criteria were meticulously adhered to for data screening: firstly, an age range was established, and individuals aged 45 years and above were included because of their elevated risk of developing diabetes. Additionally, these individuals conformed to the study's definition of middle-aged and older adults. In order to ensure that the study population was all potentially at risk of new-onset diabetes mellitus, individuals with established diabetes mellitus at baseline were excluded. Individuals afflicted with severe hepatic and renal dysfunction, malignancy and other serious chronic diseases that may interfere with lipid metabolism and inflammatory status were excluded from the study, since these may potentially affect the calculation of RCII and the analysis of associations with diabetes onset. In order to ensure the completeness and validity of the data for subsequent analyses, it was necessary to exclude data records that were incomplete or missing information. The final sample size included in the CHARLS database analysis was 9790 (Figure 1).

The ELSA study is a longitudinal research project examining the process of ageing in individuals aged 50 years and above in England [14]. For the ELSA database, the data selection criteria were equally stringent. Participants were selected on the basis of their age, with individuals aged 50 years and over forming the study group. Individuals with pre-existing diabetes at baseline and those with serious health problems were excluded from the study. It was decided that records exhibiting significant missing data would be excluded from the study. The final sample size included in the ELSA database analysis comprised 1516 individuals (Figure 1). In the context of the present study, the data set for the CHARLS was analysed, with the study period spanning from Wave 1 to Wave 5. Wave 1 was demarcated as the baseline for the analysis. For the ELSA study, data from Wave 2 to Wave 10 was analysed, with Wave 2 serving as the baseline.

### 2.2. Calculation of RCII and Determination of Diabetes

The remnant cholesterol (RC) was calculated using the following formula: RC (mg/dL) = total cholesterol (TC) − low − density lipoprotein cholesterol (LDL − C) − high − density lipoprotein cholesterol (HDL − C). RCII = RC × hsCRP (mg/L)/10 [11, 12].

Patients with diabetes included in this study could be diagnosed according to any of the following criteria: (1) fasting blood glucose (FBG) ≥7 mmol/L; (2) HbA1c level ≥ 6.5%; and (3) self-reported diagnosis of diabetes.

### 2.3. Data Collection

All basic clinical information was collected in the CHARLS and ELSA databases. A standardised questionnaire was utilised to obtain the demographic information of the participants, encompassing age, gender, marital status, educational attainment and smoking habits. Additionally, the study encompassed the assessment of comorbid chronic conditions, including hypertension and cardiovascular disease, along with anthropometric measures such as height and weight. From these data, the body mass index (BMI) was calculated. The lipid indices encompassed in this study included triglycerides (TG), TC, LDL-c and HDL-c. The following glycaemic indices are employed: FBG and glycated haemoglobin (HbA1c). Further indicators include haemoglobin and hsCRP.

### 2.4. Statistical Analysis

For the purpose of description, the mean ± standard deviation or the median (interquartile spacing) was utilised when the continuous variables were concerned. For categorical variables, frequencies and percentages were described. Cox regression analyses were conducted to explore the risk factors influencing the development of diabetes in middle-aged and older adults. Following a series of multivariate analyses, the factors identified as potentially influencing the relationship between RCII and new-onset diabetes were incorporated into the multivariate model for correction. Multivariate logistic regression analysis was employed to calculate the hazard ratio (HR) of the RCII and its 95% confidence interval (95% CI). This was done to assess the independent predictive value of the RCII for new-onset diabetes mellitus in middle-aged and elderly people, after adjusting for confounders. In order to further explore the heterogeneity of the predictive value of RCII for new-onset diabetes in middle-aged and older adults, subgroup analyses were performed. Subgroups were delineated on the basis of gender (male and female), age group (< 60 years and ≥ 60 years), BMI (< 28 and ≥ 28) and chronic disease status (hypertension prevalence, cardiovascular disease prevalence and so on). Subsequent regression analyses were executed within each subgroup in order to compare the differences observed in the relationship between RCII and new-onset diabetes in different subgroups, thereby allowing for a more in-depth investigation of the predictive efficacy of RCII under differing population characteristics and health states.

The predictive efficacy of the RCII in predicting the development of diabetes in individuals of middle and older age groups was evaluated through the utilisation of subject operating characteristic (ROC) curves. The area under the curve (AUC) of the RCII was compared with the AUC of RC and hsCRP. The objective was to evaluate the strengths and weaknesses of the RCII in predicting new-onset diabetes mellitus in middle-aged and elderly people, in comparison with traditional indicators. Additionally, all subjects were categorised according to gender, with the aim of investigating whether RCII could serve as a predictive indicator of new-onset diabetes in diverse gender demographics. For the statistical analysis, R 4.0.3 software was used. The data processing and analysis was conducted using this software. The level of statistical significance was set as *p* < 0.05.

## 3. Result

### 3.1. Baseline Characteristics of Individuals With Non-Diabetes and Diabetes

The present study comprised a total of 9946 participants from the CHARLS cohort (*n* = 9790) and ELSA cohort (*n* = 1516) (Table 1). A comparison of the age of the diabetic group in the CHARLS cohort with that of the non-diabetic participants revealed a slight decrease in the former (*p* = 0.026). However, no significant difference was observed between the two groups in the ELSA cohort (*p* = 0.351). A greater proportion of females in the CHARLS cohort developed diabetes (*p* < 0.001), whereas a higher proportion of males in the ELSA cohort were identified as having diabetes (*p* = 0.099). The proportion of smokers was higher in the diabetes group in CHARLS cohorts (*p* < 0.05). No statistically significant disparities were observed in the distribution of marital status or educational attainment between subjects with disparate glycaemic statuses within either cohort. The results of the study demonstrated that BMI, FBG and HbA1c levels were significantly higher in the diabetic group compared with the normoglycaemic group in both cohorts (all *p* < 0.001). With regard to the subject of lipid metabolism, patients in the diabetic group exhibited elevated TG levels and diminished HDL-c concentrations (*p* < 0.001). Conversely, TC and LDL-c levels demonstrated marginal increases in the CHARLS cohort, while no significant disparities were observed in the ELSA cohort. The hsCRP, RC and RCII levels were found to be significantly elevated in the diabetic group relative to the normoglycaemic group in both the CHARLS and ELSA databases, with a higher proportion observed in the high RCII quartile (*p* < 0.001). Levels of haemoglobin were found to be significantly elevated in the diabetic cohort of the ELSA study (*p* = 0.002). A near-significant difference was observed in the CHARLS cohort (*p* = 0.028). The prevalence of hypertension was found to be significantly higher in the diabetes group (*p* < 0.001). With regard to cardiovascular disease, the CHARLS cohort demonstrated a higher prevalence in the diabetes group (*p* < 0.001), while the ELSA cohort exhibited no significant difference between the two groups.

### 3.2. Univariate Cox Regression Analysis

The application of a one-way logistic regression analysis to the CHARLS and ELSA cohorts yielded findings that exhibited a significantly lower risk for females than for females in the CHARLS cohort (*p* < 0.001, HR = 1.35). The present study found no significant association between marital status and risk in the ELSA and CHARLS cohorts. The ELSA cohort revealed that females with a high school education had a 53% lower risk (*p* = 0.049, HR = 0.47). In the CHARLS cohort, males with a college education demonstrated an 81% elevated risk (*p* = 0.001, HR = 1.81). Among participants in the ELSA database, those without hypertension exhibited a significantly elevated risk, with a relative risk of 2.13 (*p* < 0.001). This finding was corroborated by the 52% risk reduction observed in the CHARLS database. There was no big change in the risk of diabetes in the ELSA group between those without CVD and those with CVD (*p* = 0.229), and a 34% reduction in the risk of diabetes in those without CVD in the CHARLS group (*p* < 0.001, HR = 0.66). For every 1 kg/m^2^ increase in BMI, the risk went up by 13% in the ELSA group (HR = 1.13); in the CHARLS group, the risk went up by 15% (HR = 1.12). The difference between the two groups was only about 1%, and both showed high significance in the whole population (*p* < 0.001). In more granular subgroup analyses, we observed that the BMI effect was marginally higher among males (HR = 1.20) than females (HR = 1.16) in the ELSA study, whereas the CHARLS study revealed comparable effects across genders (HR = 1.12 for males, HR = 1.12 for females). In ELSA, the HR for FBG was 2.65 for men and 3.86 for women, showing that the effect was stronger in women. In CHARLS, the HR was 1.01 for both sexes, and there was no difference in the strength of the effect between the sexes.

The risk in RCII quartile 4 was found to be 8.49 times higher than that in quartile 1 in the ELSA cohort (*p* < 0.001), reaching 11.00 times in males and 8.49 times in females. The corresponding HRs in CHARLS were 3.10, 2.81 and 3.45. The slope of the risk progression in the ELSA cohort was found to be significantly higher than that in CHARLS, especially in the male cohort (ELSA male HR = 11.00 vs. CHARLS male HR = 2.81). In the ELSA cohort, each 1 mg/dL increase in RC value was associated with a 1% rise in risk, whereas in CHARLS, this increase was 2%. Furthermore, the association between hsCRP and the risk of outcome was investigated. In the ELSA cohort, a significantly higher risk of outcome was observed for every 1 mg/L increase in hsCRP in the whole population (*p* < 0.001, HR = 1.22). In the context of gender stratification, a substantial degree of correlation was identified among both male and female subjects (male: *p* < 0.001, OR = 1.09; female: *p* = 0.008, OR = 1.05), with the magnitude of the observed effect exhibiting a marginal increase in its intensity among male subjects in comparison with female subjects. However, subsequent analysis revealed that hsCRP failed to demonstrate a statistically significant association with the outcome in the CHARLS cohort, considering the aggregate population (*p* = 0.183). In the context of gender stratification, a significant association was observed exclusively among the female population (*p* < 0.001, HR = 1.11); however, no such association was detected in the male population (*p* = 0.360). The remaining findings are displayed in Tables 2 and 3.

### 3.3. Subgroup Analysis

Subgroup analyses were conducted to examine the influence of demographic factors (age, sex, BMI, marriage, education and smoking) and comorbidities (hypertension and cardiovascular disease) on potential effects. In the CHARLS study, the correlation between RCII and the occurrence of diabetes was found to be more pronounced among non-obese individuals (*p* for interaction = 0.012). In the ELSA cohort, a statistically significant association between RCII and the risk of diabetes was observed in the middle-aged population (*p* for interaction = 0.048) (Table 4).

### 3.4. Association Between RCII and Diabetes

RCII was divided into four groups, with one serving as the reference group. Results indicated that within the CHARLS cohort, the HR for diabetes progressively increased as the RCII grouping rose from Group 2 to Group 4, across all participants, males and females, with all associations being statistically significant (*p* < 0.05). Specifically, among all participants, the HR (95% CI) for RCII group 4 was 3.10 (2.44–3.92) in Model 1, 2.88 (2.26–3.66) in Model 2 and 2.74 (2.15–3.49) in Model 3. Among males, the HR (95% CI) for the RCII 4 group was 2.81 (1.94–4.06) in Model 1, 2.73 (1.87–3.99) in Model 2 and 2.27 (1.48–3.50) in Model 3. In the female cohort, the HR (95% CI) for the RCII 4 group was 3.30 (2.42–4.49) in Model 1, 2.92 (2.14–3.99) in Model 2 and 2.40 (1.68–3.42) in Model 3 (Table 5).

Results indicated that within the ELSA cohort, the HR for diabetes progressively increased as the RCII grouping rose from Group 2 to Group 4, across all participants, males and females, with all associations being statistically significant (*p* < 0.05). Specifically, among all participants, the HR (95% CI) for RCII group 4 was 8.49 (4.39–16.41) in Model 1, 9.17 (4.66–18.05) in Model 2 and 7.13 (3.49–14.53) in Model 3. Among males, the HR (95% CI) for the RCII 4 group was 11.00 (4.24–28.53) in Model 1, 9.89 (3.79–25.82) in Model 2 and 8.10 (2.96–22.13) in Model 3. In the female cohort, the HR (95% CI) for the RCII 4 group was 8.49 (3.27–22.06) in Model 1, 7.93 (3.03–20.71) in Model 2 and 5.72 (2.08–15.75) in Model 3 (Table 6).

## 4. Discussion

In the present study, both the CHARLS and ELSA cohorts demonstrated a significant and positive correlation between RCII and the risk of diabetes in middle-aged and older adults. The stability of this association was confirmed through the implementation of adjustments for confounding factors, including sex, smoking, hypertension and other potential confounders. This finding is consistent with the results of previous studies, which demonstrated that RC and inflammatory factors are independently implicated in the pathophysiology of diabetes. Research has demonstrated a correlation between RC and the progression of diabetes, with the potential to exacerbate atherosclerosis and insulin resistance. Elevated CRP, a hallmark of inflammation, has been linked to impaired *β*-cell function [15–17]. Furthermore, the higher sensitivity of hsCRP to validation in comparison with CRP is indicative of its greater importance in the pathogenesis of various metabolic diseases [18–20]. Furthermore, the presence of prevalent metabolic abnormalities in middle-aged and older adults, including elevated BMI, FBG and HbA1c, was more pronounced in patients with diabetes. This provides a metabolic background for the pathogenic mechanism of RCII.

The present study corroborates the hypothesis that the RCII is superior in diabetes prediction. This is evidenced by the demonstration that a combination of markers is a more accurate reflection of the complex pathology of the disease than a single indicator, such as all-cause mortality and cardiovascular disease convenience. A plethora of studies on metabolic syndrome have previously indicated that the combination of lipid and inflammatory markers can enhance the precision of cardiovascular event prediction, and the present study extends this concept to diabetes. RC, as triglyceride-rich lipoprotein residues, has been demonstrated to induce insulin resistance and *β*-cell lipotoxicity through deposition in the vessel wall and pancreas. Previous studies have also established the association of RC with a variety of metabolic diseases [21–23]. Furthermore, hsCRP activates the nuclear factor-*κ*B (NF-*κ*B) pathway, inhibits insulin signalling, and promotes the secretion of pro-inflammatory factors by adipocytes, exacerbating glucose metabolism disorders [24]. The present study demonstrated that both hsCRP and RC levels were significantly higher in patients with diabetes than in non-diabetic patients, thereby supporting the synergistic effect of inflammation and lipid metabolism disorders.

A comparison of the CHARLS and ELSA databases reveals the extreme effect of FBG and HbA1C in the ELSA cohort. This finding indicates that the population may be associated with more severe cases of glucose metabolism disorders and a higher proportion of advanced diabetes cases in the samples. Potential explanations for these observations include differences in assay methodology or genetic background of the population. Conversely, the results of the CHARLS are more closely related to metabolic profiles of middle-aged and elderly populations in general. The evidence presented indicated an association exclusively among women, which may be attributable to variations in female hormone levels or immune responses. The overall association of hsCRP in ELSA may be indicative of an elevated inflammatory baseline within the population. Both cohorts demonstrated a higher risk of diabetes in non-smokers, which contradicts conventional wisdom and may be attributable to the following factors: the non-smoking group may have included more passive quitters because of disease or a population with worse baseline health; the univariate analysis did not adjust for variables such as age, BMI and disease history; and smoking may be co-varied with other protective factors (e.g., alcohol consumption, exercise). Furthermore, smoking patterns exhibited significant variation between ELSA and CHARLS, a factor that may have resulted in effect heterogeneity. However, in the multivariate model, the correction for smoking factors did not affect the risk of diabetes onset by RCII.

The present study revealed a stronger correlation between RCII and diabetes in the ELSA cohort compared with the CHARLS cohort. Potential explanations for this discrepancy may include the following factors: Firstly, there are disparities in population characteristics. The ELSA cohort exhibited significantly higher BMI and RC levels compared with CHARLS, indicating that the elevated baseline prevalence of metabolic disorders observed in Western populations may have amplified the impact of RCII. Secondly, there are discrepancies in the assays employed. ELSA utilised hsCRP, while CHARLS may have employed conventional CRP. The use of hsCRP in ELSA, in comparison with conventional CRP in CHARLS, is indicative of a heightened sensitivity to low-level inflammation, which has been demonstrated to augment the predictive capacity of RCII. Thirdly, the period of observation was found to be considerably lengthier in the ELSA cohort than in the CHARLS cohort. This phenomenon is attributed to a cumulative effect of time that was more pronounced in ELSA. However, the findings of this study also indicate that early intervention in middle-aged and elderly adults with elevated RCII levels may significantly reduce the future risk of developing diabetes. This approach also facilitates the early identification of high-risk individuals, enabling timely intervention. In addition, the clinical accessibility of RCII renders it suitable for routine screening in this population, thereby holding significant potential for reducing public health expenditure.

Research has demonstrated that both independent and synergistic effects are exerted by RC, TG and inflammatory markers in the prediction of diabetes. There is a robust body of evidence to suggest that persistently elevated RC levels are significantly associated with an increased risk of diabetes. For instance, cohort studies such as CHARLS and ELSA demonstrate that individuals with sustained high RC levels face a 1.98- to 2.73-fold increased risk of diabetes, independent of LDL-C levels [17]. A nationwide Korean cohort study further confirmed that each quartile increase in RC elevates diabetes risk by 25%–95%, with particularly pronounced predictive value in populations with fewer traditional risk factors [25]. It is evident that TG play a pivotal role in the pathogenesis of diabetes, given their correlation with both insulin resistance and lipid metabolism disorders. Elevated TG levels have been found to correlate positively with diabetes risk, whereas extremely low TG levels may exhibit a non-linear association because of potential disruption of hepatic glucose regulation mechanisms, thereby increasing risk [26]. CRP has been demonstrated to mediate chronic low-grade inflammation, thereby promoting insulin resistance and *β*-cell dysfunction. Prospective studies have indicated that elevated CRP levels increase diabetes risk by 2- to 3-fold, with higher predictive sensitivity observed for hsCRP [27, 28]. Elevated RC and TG levels frequently co-occur with dyslipidaemia and inflammatory activation, forming a vicious cycle where inflammation exacerbates lipid deposition and insulin resistance. The 2025 ADA guidelines have designated non-HDL-C as a secondary target and recommended CRP monitoring to optimise diabetes risk assessment. In summary, these three indicators independently or synergistically predict diabetes through distinct pathological pathways. The combination of these assessments has been shown to significantly enhance their predictive efficacy, thereby providing a solid foundation for the implementation of early intervention strategies.

In the context of metabolic syndrome, RC has been identified as a significant contributor to the development of insulin resistance, with the capacity to accumulate in insulin-sensitive organs such as the pancreas and adipose tissue. The subsequent accumulation of these cells may lead to the activation of macrophages, resulting in a shift towards a pro-inflammatory phenotype. This process is accompanied by the release of inflammatory mediators, such as tumour necrosis factor-*α* (TNF-*α*) and interleukin-6 (IL-6), which collectively establish a chronic pro-inflammatory lipid microenvironment. This process is comprehensively reflected by RCII. Within the pancreas, pro-inflammatory factors directly impair *β*-cell insulin secretion granule synthesis and release via the NF-*κ*B signalling pathway, concurrently inducing *β*-cell apoptosis, resulting in absolute insulin deficiency. In the liver, skeletal muscle, and adipose tissue, inflammatory mediators inhibit the phosphorylation of insulin receptor substrate (IRS), thus disrupting insulin signalling. This, in combination with the abnormal release of free fatty acids induced by residual cholesterol, further exacerbates insulin resistance. The synergistic interaction between the aforementioned *β*-cell dysfunction and insulin resistance ultimately disrupts the body's glucose metabolic homeostasis, significantly increasing the risk of diabetes onset. This finding indicates that RCII is more likely to be a characteristic marker of a chronic pro-inflammatory lipid environment. It functions indirectly in the pathological process of diabetes by mediating the cascade effects of lipid metabolism disorders and inflammatory responses, rather than acting as a direct causative agent.

It is important to note that the study is not without its limitations. Firstly, the relatively limited sample size of the elderly population in the ELSA cohort, in conjunction with the inconsistency of follow-up time between the two cohorts, has the potential to compromise the evaluation of the long-term predictive efficacy of RCII. Secondly, the present study did not consider diet, exercise and other confounding factors affecting the risk of developing diabetes in middle-aged and older adults. It is possible that these factors may have an impact on the results and therefore require further elucidation in more detailed studies in the future. It is evident that, despite the similarities in design between CHARLS and ELSA as cohort studies, there remains significant heterogeneity between the two datasets. Furthermore, the ELSA data exhibited a high attrition rate during follow-up, which constitutes a major limitation of this study. Notwithstanding, consistent results were observed in these two independent analyses, thereby enhancing the generalisability and applicability of the results of this study.

## 5. Conclusion

The present study demonstrated that RCII is strongly associated with the development of diabetes in a cohort of middle-aged and older adults. This finding provides a new tool for early risk assessment of diabetes in middle-aged and elderly people, and suggests that monitoring and intervention of RCII levels in these groups should be emphasised in clinical practice to reduce the risk of diabetes.

## Figures and Tables

**Table 1:** Baseline characteristics of included participants.

**Variables**	**CHARLS**				**ELSA**			
	**Total (** **n** = 9790**)**	**Normal (** **n** = 9083**)**	**Diabetes (** **n** = 707**)**	**p**	**Total (** **n** = 1516**)**	**Normal (** **n** = 1370**)**	**Diabetes (** **n** = 146**)**	**p**
Age (y)	59.30 ± 9.58	59.35 ± 9.67	58.62 ± 8.32	0.026	60.51 ± 5.88	60.56 ± 5.86	60.08 ± 6.10	0.351
BMI (kg/m^2^)	23.35 ± 3.76	23.19 ± 3.71	25.37 ± 3.83	< 0.001	27.37 ± 4.28	27.04 ± 4.12	30.42 ± 4.57	< 0.001
FBG (mg/dL)	106.32 ± 26.39	104.53 ± 22.88	129.35 ± 48.45	< 0.001	4.91 ± 0.56	4.86 ± 0.51	5.30 ± 0.85	< 0.001
TC (mg/dL)	193.10 ± 37.51	192.58 ± 37.36	199.76 ± 38.70	< 0.001	236.54 ± 43.54	236.51 ± 42.78	236.81 ± 50.28	0.945
HDL-c (mg/dL)	51.43 ± 14.91	51.85 ± 14.96	46.12 ± 13.19	< 0.001	60.98 ± 17.74	61.80 ± 18.06	53.31 ± 12.01	< 0.001
LDL-c (mg/dL)	117.04 ± 34.05	116.69 ± 33.81	121.53 ± 36.67	< 0.001	135.04 ± 67.51	136.60 ± 63.31	120.34 ± 97.66	0.051
TG (mg/dL)	105.32 (76.11, 153.10)	103.54 (74.34, 149.57)	140.71 (96.46, 203.11)	< 0.001	124.04 (88.60, 186.06)	124.04 (88.60, 177.20)	177.20 (126.25, 263.59)	< 0.001
HbA1c (%)	5.18 ± 0.63	5.14 ± 0.53	5.79 ± 1.23	< 0.001	5.42 ± 0.45	5.37 ± 0.31	5.95 ± 0.94	< 0.001
Haemoglobin (g/L)	14.41 ± 2.22	14.40 ± 2.22	14.59 ± 2.23	0.028	14.45 ± 1.50	14.41 ± 1.53	14.82 ± 1.15	0.002
hsCRP (mg/L)	0.96 (0.53, 1.91)	0.94 (0.53, 1.87)	1.25 (0.65, 2.30)	< 0.001	1.50 (0.70, 2.90)	1.40 (0.70, 2.70)	2.75 (1.42, 4.40)	< 0.001
RC (mg/dL)	19.72 (11.60, 31.70)	19.33 (11.21, 30.93)	26.68 (16.62, 41.17)	< 0.001	23.22 (19.35, 38.70)	23.22 (19.35, 37.73)	34.83 (24.19, 50.31)	0.002
RCII	1.91 (0.81, 4.67)	1.84 (0.79, 4.49)	3.26 (1.35, 7.50)	< 0.001	4.06 (1.74, 9.06)	3.72 (1.55, 8.12)	9.71 (4.63, 18.34)	< 0.001
RCII quantile, *n* (%)				< 0.001				< 0.001
1	2423 (24.75)	2331 (25.66)	92 (13.01)		378 (24.93)	368 (26.86)	10 (6.85)	
2	2457 (25.10)	2307 (25.40)	150 (21.22)		378 (24.93)	358 (26.13)	20 (13.70)	
3	2461 (25.14)	2267 (24.96)	194 (27.44)		379 (25.00)	340 (24.82)	39 (26.71)	
4	2449 (25.02)	2178 (23.98)	271 (38.33)		381 (25.13)	304 (22.19)	77 (52.74)	
Gender, *n* (%)				< 0.001				0.099
Male	4614 (47.13)	4331 (47.68)	283 (40.03)		681 (44.92)	606 (44.23)	75 (51.37)	
Female	5176 (52.87)	4752 (52.32)	424 (59.97)		835 (55.08)	764 (55.77)	71 (48.63)	
Marry, *n* (%)				0.048				0.375
Married or partnered	8084 (82.57)	7481 (82.36)	603 (85.29)		1166 (76.91)	1058 (77.23)	108 (73.97)	
Other status	1706 (17.43)	1602 (17.64)	104 (14.71)		350 (23.09)	312 (22.77)	38 (26.03)	
Education, *n* (%)				0.908				0.974
Below high school	4622 (47.21)	4283 (47.15)	339 (47.95)		635 (41.89)	573 (41.82)	62 (42.47)	
High school	4118 (42.06)	3826 (42.12)	292 (41.30)		375 (24.74)	340 (24.82)	35 (23.97)	
College or above	1050 (10.73)	974 (10.72)	76 (10.75)		506 (33.38)	457 (33.36)	49 (33.56)	
Smoke, *n* (%)				< 0.001				0.071
Never smokers	3851 (39.35)	3621 (39.88)	230 (32.53)		1362 (89.90)	1237 (90.36)	125 (85.62)	
Ever smokers	5935 (60.65)	5458 (60.12)	477 (67.47)		153 (10.10)	132 (9.64)	21 (14.38)	
Hypertension, *n* (%)				< 0.001				< 0.001
Yes	2315 (23.73)	2055 (22.71)	260 (36.83)		1340 (88.39)	1225 (89.42)	115 (78.77)	
No	7441 (76.27)	6995 (77.29)	446 (63.17)		176 (11.61)	145 (10.58)	31 (21.23)	
Cardiovascular disease, *n* (%)				< 0.001				0.217
Yes	1103 (11.31)	991 (10.95)	112 (15.91)		160 (14.45)	154 (14.78)	6 (9.23)	
No	8650 (88.69)	8058 (89.05)	592 (84.09)		947 (85.55)	888 (85.22)	59 (90.77)	

*Note:* Comparison of baseline clinical characteristics between patients with diabetic and non-diabetic patients at the end of follow-up.

Abbreviations: BMI, body mass index; FBG: fasting blood glucose; HbA1c: glycated haemoglobin; HDL-c: high-density lipoprotein cholesterol; hsCRP: high-sensitivity C-reactive protein, LDL-c: low-density lipoprotein cholesterol; RC: remnant cholesterol; RCII: remnant cholesterol inflammatory index; TC: total cholesterol; TG: triglyceride.

**Table 2:** Univariate cox regression analysis in CHARLS.

**Variables**	**All participants**		**Male**		**Female**	
	**p**	**HR (95% CI)**	**p**	**HR (95% CI)**	**p**	**HR (95% CI)**
Gender						
Male		1.00 (Reference)				
Female	< 0.001	1.35 (1.16–1.57)				
Marry						
Married or partnered		1.00 (Reference)		1.00 (Reference)		1.00 (Reference)
Other status	0.050	0.81 (0.66–0.99)	0.003	0.52 (0.33–0.80)	0.500	0.92 (0.72–1.17)
Education						
Below high school		1.00 (Reference)		1.00 (Reference)		1.00 (Reference)
High school	0.670	0.97 (0.83–1.13)	0.018	1.40 (1.06–1.85)	0.440	0.92 (0.75–1.13)
College or above	0.934	0.99 (0.77–1.27)	0.001	1.81 (1.2–2.57)	0.041	0.64 (0.41–0.98)
Smoke						
Yes		1.00 (Reference)		1.00 (Reference)		1.00 (Reference)
No	< 0.001	1.36 (1.16–1.59)	0.123	1.22 (0.95–1.58)	0.502	1.14 (0.78–1.66)
Hypertension						
Yes		1.00 (Reference)		1.00 (Reference)		1.00 (Reference)
No	< 0.001	0.52 (0.45–0.61)	< 0.001	0.60 (0.46–0.76)	< 0.001	0.48 (0.40–0.59)
Cardiovascular disease						
Yes		1.00 (Reference)		1.00 (Reference)		1.00 (Reference)
No	< 0.001	0.66 (0.54–0.81)	< 0.001	0.55 (0.40–0.76)	0.046	0.77 (0.59–0.99)
RCII quantile						
1		1.00 (Reference)		1.00 (Reference)		1.00 (Reference)
2	< 0.001	1.62 (1.25–2.10)	0.005	1.75 (1.18–2.58)	0.018	1.52 (1.08–2.16)
3	< 0.001	2.12 (1.66–2.72)	0.009	1.69 (1.14–2.50)	< 0.001	2.49 (1.81–3.43)
4	< 0.001	3.10 (2.44–3.92)	< 0.001	2.81 (1.94–4.06)	< 0.001	3.45 (2.53–4.69)
Age (y)	0.054	0.99 (0.98–1.00)	< 0.001	0.98 (0.96–0.99)	0.402	1.00 (0.99–1.01)
BMI (kg/m^2^)	< 0.001	1.12 (1.10–1.14)	< 0.001	1.12 (1.09–1.15)	< 0.001	1.12 (1.09–1.14)
FBG (mg/dL)	< 0.001	1.01 (1.01–1.01)	< 0.001	1.01 (1.01–1.01)	< 0.001	1.01 (1.01–1.01)
HDL-c (mg/dL)	< 0.001	0.97 (0.97–0.98)	< 0.001	0.97 (0.96–0.98)	< 0.001	0.97 (0.96–0.98)
TC (mg/dL)	< 0.001	1.01 (1.01–1.01)	0.182	1.00 (1.00–1.01)	< 0.001	1.01 (1.01–1.01)
LDL-c (mg/dL)	< 0.001	1.01 (1.01–1.01)	0.640	1.00 (1.00–1.00)	< 0.001	1.01 (1.01–1.01)
TG (mg/dL)	< 0.001	1.01 (1.01–1.01)	< 0.001	1.01 (1.01–1.01)	< 0.001	1.01 (1.01–1.01)
hsCRP (mg/L)	0.183	1.08 (1.04–1.12)	0.360	1.03 (0.97–1.10)	< 0.001	1.11 (1.06–1.17)
HAb1c (%)	< 0.001	1.78 (1.71–1.86)	< 0.001	1.77 (1.64–1.90)	< 0.001	1.78 (1.69–1.88)
Haemoglobin (g/L)	0.027	1.04 (1.01–1.07)	0.030	1.06 (1.01–1.11)	0.002	1.07 (1.02–1.11)
RC (mg/dL)	< 0.001	1.02 (1.01–1.02)	< 0.001	1.02 (1.01–1.02)	< 0.001	1.01 (1.01–1.02)

Abbreviations: BMI, body mass index; FBG: fasting blood glucose; HbA1c: glycated haemoglobin; HDL-c: high-density lipoprotein cholesterol; hsCRP: high-sensitivity C-reactive protein, LDL-c: low-density lipoprotein cholesterol; RC: remnant cholesterol; RCII: remnant cholesterol inflammatory index; TC: total cholesterol; TG: triglyceride.

**Table 3:** Univariate cox regression analysis in ELSA.

**Variables**	**All participants**		**Male**		**Female**	
	**p**	**OR (95% CI)**	**p**	**OR (95% CI)**	**p**	**OR (95% CI)**
Gender						
Male		1.00 (Reference)				
Female	0.100	0.76 (0.55–1.05)				
Marry						
Married or partnered		1.00 (Reference)		1.00 (Reference)		1.00 (Reference)
Other status	0.534	1.13 (0.77–1.64)	0.271	1.37 (0.78–2.40)	0.736	1.09 (0.65–1.83)
Education						
Below high school		1.00 (Reference)		1.00 (Reference)		1.00 (Reference)
High school	0.773	0.94 (0.62–1.42)	0.154	1.46 (0.87–2.48)	0.049	0.47 (0.22–0.99)
College or above	0.940	0.99 (0.68–1.43)	0.894	1.04 (0.59–1.84)	0.999	1.00 (0.60–1.66)
Smoke						
Yes		1.00 (Reference)		1.00 (Reference)		1.00 (Reference)
No	0.029	1.67 (1.06–2.65)	0.033	1.99 (1.06–3.76)	0.296	1.43 (0.73–2.83)
Hypertension						
Yes		1.00 (Reference)		1.00 (Reference)		1.00 (Reference)
No	< 0.001	2.13 (1.43–3.17)	< 0.001	2.62 (1.52–4.51)	0.081	1.80 (0.93–3.49)
Cardiovascular disease						
Yes		1.00 (Reference)		1.00 (Reference)		1.00 (Reference)
No	0.229	1.67 (0.72–3.88)	0.113	3.23 (0.76–13.72)	0.994	1.00 (0.34–2.96)
RCII quantile						
1		1.00 (Reference)		1.00 (Reference)		1.00 (Reference)
2	0.070	2.02 (0.94–4.31)	0.040	2.99 (1.05–8.48)	0.160	2.16 (0.74–6.32)
3	< 0.001	4.01 (2.00–4.31)	< 0.001	5.32 (1.98–14.28)	0.002	4.67 (1.73–12.56)
4	< 0.001	8.49 (4.39–16.40)	< 0.001	11.00 (4.24–28.53)	< 0.001	8.49 (3.27–22.06)
Age (y)	0.356	0.99 (0.96–1.02)	0.229	0.98 (0.94–1.02)	0.260	0.98 (0.93–1.02)
BMI (kg/m^2^)	< 0.001	1.13 (1.10–1.66)	< 0.001	1.20 (1.14–1.27)	< 0.001	1.16 (1.11–1.21)
FBG (mg/dL)	< 0.001	2.77 (2.17–3.55)	< 0.001	2.65 (1.74–4.04)	< 0.001	3.86 (2.38–6.26)
HDL-c (mg/dL)	< 0.001	0.99 (0.99–0.99)	0.036	0.98 (0.96–0.99)	< 0.001	0.95 (0.94–0.97)
TC (mg/dL)	0.904	1.00 (1.00–1.00)	0.946	1.00 (0.99–1.01)	0.407	1.00 (1.00–1.01)
LDL-c (mg/dL)	0.003	0.99 (0.99–0.99)	0.003	0.99 (0.99–0.99)	0.425	1.00 (0.99–1.00)
TG (mg/dL)	< 0.001	1.01 (1.01–1.01)	< 0.001	1.01 (1.01–1.01)	< 0.001	1.01 (1.01–1.01)
hsCRP (mg/L)	< 0.001	1.22 (1.15–1.30)	< 0.001	1.09 (1.04–1.13)	0.008	1.05 (1.01–1.10)
HAb1c (%)	< 0.001	2.11 (1.91–2.33)	< 0.001	27.35 (12.38–60.44)	< 0.001	115.31 (43.07–308.70)
Haemoglobin (g/L)	< 0.001	1.24 (1.09–1.40)	0.060	1.22 (0.99–1.50)	0.026	1.27 (1.03–1.56)
RC (mg/dL)	< 0.001	1.01 (1.01–1.01)	0.001	1.01 (1.01–1.01)	0.011	1.01 (1.01–1.01)

Abbreviations: BMI, body mass index; FBG: fasting blood glucose; HbA1c: glycated haemoglobin; HDL-c: high-density lipoprotein cholesterol; hsCRP: high-sensitivity C-reactive protein, LDL-c: low-density lipoprotein cholesterol; RC: remnant cholesterol; RCII: remnant cholesterol inflammatory index; TC: total cholesterol; TG: triglyceride.

**Table 4:** Subgroup analysis of the relationship between RCII and diabetes.

**Variables**	**CHARLS**			**ELSA**		
	**HR (95% CI)**	**p**	**p** ** for interaction**	**HR (95% CI)**	**p**	**p** ** for interaction**
All patients	1.03 (1.02–1.04)	< 0.001		1.01 (1.01–1.01)	< 0.001	
Gender			0.660			0.191
Male	1.03 (1.02–1.04)	< 0.001		1.01 (1.00–1.01)	< 0.001	
Female	1.03 (1.02–1.04)	< 0.001		1.01 (1.01–1.02)	< 0.001	
Marry			0.156			0.866
Married or partnered	1.03 (1.02–1.04)	< 0.001		1.01 (1.00–1.01)	< 0.001	
Other status	1.01 (0.99–1.04)	0.188		1.01 (1.01–1.01)	< 0.001	
Education			0.560			0.337
Below high school	1.03 (1.02–1.04)	< 0.001		1.01 (1.01–1.01)	< 0.001	
High school	1.03 (1.02–1.04)	< 0.001		1.00 (1.00–1.01)	0.339	
College or above	1.04 (1.02–1.05)	< 0.001		1.01 (1.01–1.02)	< 0.001	
Smoke			0.746			0.840
Yes	1.03 (1.02–1.04)	< 0.001		1.01 (1.01–1.01)	< 0.001	
No	1.03 (1.02–1.04)	< 0.001		1.01 (0.99–1.02)	0.260	
Hypertension			0.216			0.655
Yes	1.02 (1.01–1.03)	< 0.001		1.01 (1.01–1.01)	< 0.001	
No	1.03 (1.02–1.04)	< 0.001		1.01 (1.00–1.02)	0.149	
Cardiovascular disease			0.072			0.933
Yes	1.02 (1.00–1.03)	0.021		1.25 (0.88–1.78)	0.205	
No	1.03 (1.02–1.04)	< 0.001		1.24 (1.11–1.38)	< 0.001	
Age			0.256			0.048
<60	1.03 (1.02–1.04)	< 0.001		1.02 (1.01–1.03)	< 0.001	
≥60	1.02 (1.01–1.03)	< 0.001		1.01 (1.00–1.01)	< 0.001	
BMI			0.012			0.239
<28	1.03 (1.02–1.04)	< 0.001		1.01 (1.01–1.01)	< 0.001	
≥28	1.01 (0.99–1.02)	0.339		1.01 (1.01–1.02)	< 0.001	

**Table 5:** Longitudinal association between RCII and diabetes in CHARLS.

**Variables**		**Model1**		**Model2**		**Model3**	
		**HR (95% CI)**	**p**	**HR (95% CI)**	**p**	**HR (95% CI)**	**p**
	RCII						
All participants	1	1.00 (Reference)		1.00 (Reference)		1.00 (Reference)	
	2	1.62 (1.25–2.10)	< 0.001	1.62 (1.25–2.11)	< 0.001	1.58 (1.21–2.05)	< 0.001
	3	2.12 (1.66–2.72)	< 0.001	2.04 (1.58–2.62)	< 0.001	1.95 (1.51–2.51)	< 0.001
	4	3.10 (2.44–3.92)	< 0.001	2.88 (2.26–3.66)	< 0.001	2.74 (2.15–3.49)	< 0.001
	RCII						
Male	1	1.00 (Reference)		1.00 (Reference)		1.00 (Reference)	
	2	1.75 (1.18–2.58)	0.005	1.80 (1.21–2.68)	0.004	1.90 (1.23–2.94)	0.004
	3	1.69 (1.14–2.50)	0.009	1.69 (1.13–2.52)	0.010	1.57 (1.01–2.46)	0.046
	4	2.81 (1.94–4.06)	< 0.001	2.73 (1.87–3.99)	< 0.001	2.27 (1.48–3.50)	< 0.001
	RCII						
Female	1	1.00 (Reference)		1.00 (Reference)		1.00 (Reference)	
	2	1.52 (1.08–2.16)	0.018	1.46 (1.03–2.07)	0.033	1.35 (0.92–2.00)	0.125
	3	2.49 (1.81–3.43)	< 0.001	2.26 (1.63–3.12)	< 0.001	2.15 (1.50–3.07)	< 0.001
	4	3.30 (2.42–4.49)	< 0.001	2.92 (2.14–3.99)	< 0.001	2.40 (1.68–3.42)	< 0.001

*Note:* Model1: Crude; Model2: Adjust: Gender, smoke, hypertension and cardiovascular disease; Model3: Adjust: Gender, smoke, hypertension, cardiovascular disease, age, triglycerides, haemoglobin and body mass index.

**Table 6:** Longitudinal association between RCII and diabetes in ELSA.

**Variables**		**Model1**		**Model2**		**Model3**	
		**HR (95% CI)**	**p**	**HR (95% CI)**	**p**	**HR (95% CI)**	**p**
	RCII						
All participants	1	1.00 (Reference)		1.00 (Reference)		1.00 (Reference)	
	2	2.02 (0.94–4.31)	0.012	2.49 (1.18–5.27)	0.017	2.35 (1.11–4.98)	0.026
	3	4.01 (2.00–4.31)	< 0.001	4.65 (2.30–9.40)	< 0.001	4.13 (2.03–8.42)	< 0.001
	4	8.49 (4.39–16.40)	< 0.001	9.17 (4.66–18.05)	< 0.001	7.13 (3.49–14.53)	< 0.001

	RCII						
Male	1	1.00 (Reference)		1.00 (Reference)		1.00 (Reference)	
	2	2.99 (1.05–8.48)	0.040	2.84 (1.00–8.11)	0.051	2.66 (0.93–7.61)	0.068
	3	5.32 (1.98–14.28)	< 0.001	5.21 (1.93–14.10)	0.001	4.56 (1.66–12.49)	0.003
	4	11.00 (4.24–28.53)	< 0.001	9.89 (3.79–25.82)	< 0.001	8.10 (2.96–22.13)	< 0.001

	RCII						
Female	1	1.00 (Reference)		1.00 (Reference)		1.00 (Reference)	
	2	2.16 (0.74–6.32)	0.160	2.16 (0.73–6.33)	0.162	2.10 (0.71–6.19)	0.181
	3	4.67 (1.73–12.56)	0.002	4.56 (1.69–12.32)	0.003	4.00 (1.46–10.95)	0.007
	4	8.49 (3.27–22.06)	< 0.001	7.93 (3.03–20.71)	< 0.001	5.72 (2.08–15.75)	< 0.001

*Note:* Model1: Crude; Model2: Adjust: Gender, smoke, hypertension and cardiovascular disease; Model3: Adjust: Gender, smoke, hypertension, cardiovascular disease, age, triglycerides, haemoglobin and body mass index.

## Data Availability

The data that support the findings of this study are available from the corresponding author upon reasonable request.
